# Effects of Different Prenatal Nutrition Strategies on the Liver Metabolome of Bulls and Its Correlation with Body and Liver Weight

**DOI:** 10.3390/metabo12050441

**Published:** 2022-05-14

**Authors:** Guilherme Henrique Gebim Polizel, Fernando Augusto Correia Queiroz Cançado, Evandro Fernando Ferreira Dias, Arícia Christofaro Fernandes, Roberta Cavalcante Cracco, Brenda Tonsic Carmona, Henrique Hespanhol Castellar, Mirele Daiana Poleti, Miguel Henrique de Almeida Santana

**Affiliations:** 1 Department of Animal Science, Faculty of Animal Science and Food Engineering–USP, Av. Duque de Caxias Norte, 225, Pirassununga 13635-900, SP, Brazil; guilhermepolizel875@gmail.com (G.H.G.P.); evandrodias4@usp.br (E.F.F.D.); aricia.fernandes@usp.br (A.C.F.); roberta.cracco@usp.br (R.C.C.); brendacarmona@usp.br (B.T.C.); henriquehcastellar@usp.br (H.H.C.); 2 Department of Basic Sciences, Faculty of Animal Science and Food Engineering–USP, Av. Duque de Caxias Norte, 225, Pirassununga 13635-900, SP, Brazil; facqc@usp.br (F.A.C.Q.C.); mirelep@usp.br (M.D.P.)

**Keywords:** beef cattle, fetal programming, hepatic metabolites, maternal nutrition

## Abstract

This study investigated the effect of prenatal nutrition on liver metabolome and on body (BW) and liver weight (LW) of Nellore bulls at slaughter. Three treatments were applied in 126 cows during pregnancy: NP—control (mineral supplementation); PP—protein-energy supplementation in the third trimester; and FP—protein-energy supplementation during the entire pregnancy. Offspring BW and LW were evaluated, and a targeted metabolomics analysis was performed on their livers (*n* = 18, 22.5 ± 1 months of age). Data were submitted to principal component analysis (PCA), analysis of variance (ANOVA), enrichment analysis, and Pearson’s correlation analysis. The phenotypes did not show differences between treatments (*p* > 0.05). Metabolites PCA showed an overlap of treatment clusters in the analysis. We found significant metabolites in ANOVA (*p* ≤ 0.05; Glycine, Hydroxytetradecadienylcarnitine, Aminoadipic acid and Carnosine). Enrichment analysis revealed some biological processes (Histidine metabolism, beta-Alanine metabolism, and Lysine degradation). Pearson’s correlation analysis showed 29 significant correlated metabolites with BW and 1 metabolite correlated with LW. In summary, prenatal nutrition did not show effects on the phenotypes evaluated, but affected some metabolites and biological pathways, mainly related to oxidative metabolism. In addition, BW seems to influence the hepatic metabolome more than LW, due to the amount and magnitude of correlations found.

## 1. Introduction

In the last two decades, maternal nutrition has been substantially discussed, given its long-term impacts on offspring [1,2,3,4,5]. In ruminants, the placentomes (caruncular-cotiledonary unit of the placenta) support nutrient exchanges between mother and fetus during pregnancy [6]. These physiological exchanges may be affected by environmental factors, such as nutrition, and impact fetal development with lifelong phenotypic and metabolic consequences [7,8,9]. In beef cattle, a large number of studies have focused mainly on evaluating the impacts of prenatal nutrition on the muscle performance of the offspring. However, only a few recent studies have assessed the effects of prenatal nutrition on the liver [10,11].

The liver is a complex organ of metabolism that play several important roles. Besides being the central organ in the energy metabolism (lipids and carbohydrates), it acts in the metabolization of bilirubin, bile acids, and xenobiotics, and in protein synthesis and immunity [12]. Several studies have reported associations between liver metabolic and biological processes and feed efficiency in beef cattle [13,14,15]. Nevertheless, the molecular mechanisms that prenatal nutrition may affect in the offspring’s liver remain largely unknown.

Metabolomics analysis uses some advanced analytical chemistry techniques to globally assess the metabolite profiles present in cells, tissues, organs, and/or in biofluids [16]. Metabolites are the last layer of the “omics” sciences, carrying imprints of genomic, transcriptomic, epigenomic, and environmental effects. In this context, scientific interest in the interaction between nutrition and metabolome has increased in recent years [17], including that related to prenatal nutrition [18,19,20].

In summary, the hypothesis of our study is that different prenatal supplementation strategies can change the liver metabolic profile of bulls. The aims of this study were: (1) To assess whether prenatal nutrition had an effect on liver weight (LW) and on the pre-slaughter body weight (BW) of bulls in the finishing phase; (2) To evaluate whether the different prenatal nutritional conditions impacted on the liver metabolome (amino acid, biogenic amines, hexose, acylcarnitines, lysophosphatidylcholines, phosphatidylcholines, and sphingolipids) of bulls through the targeted metabolomics approach; (3) To perform Pearson’s correlations analyzes to assess the association of BW and LW with hepatic metabolites.

## 2. Results

### 2.1. Pre-Slaughter Body Weight and Liver Weight

The different prenatal supplementation strategies (NP, PP, and FP) had no effect on pre-slaughter BW (*p* = 0.685) and LW (*p* = 0.924) in bulls at the end of the finishing phase (Table 1).

### 2.2. Univariate Analysis of Liver Metabolome

In the liver metabolomics assessment, the bulls presented four metabolites (Table 2) expressed differentially among the groups (Glycine (Gly) [*p* = 0.024]; Hydroxytetradecadienylcarnitine (C14:2-OH) [*p* = 0.042]; Aminoadipic acid (Alpha-aaa) [*p* = 0.043]; Carnosine [*p* = 0.050]). The FP treatment showed the lowest concentrations of C14:2-OH and Carnosine compared to the NP treatment. The NP group showed no difference between the other treatments for Gly; however, FP showed a higher concentration of this metabolite in comparison with the PP treatment. Regarding Alpha-aaa, the FP treatment showed lower concentrations than the other two groups. The tables with all metabolites (significant and not significant) and respective *p*-values can be found in the Supplementary Material (Table S1).

### 2.3. Principal Component Analysis (PCA)

**Figure 1:**
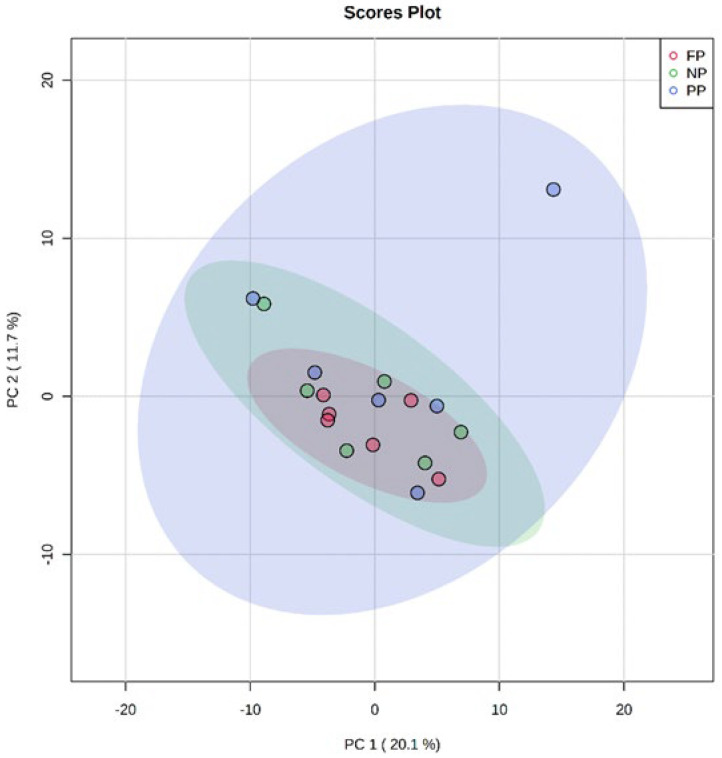
Principal component analysis (PCA) scores plot of metabolome distribution of bulls’ liver among the treatments (NP, PP, and FP).

According to the results found in PCA of the fetal programming in bulls (Figure 1), the distribution of data showed an overlap between all groups, and it was not possible to observe a clustering among the treatments. This may indicate that the metabolite profile presented only a few or non-variables expressed differentially among treatments. The two principal components together explain 31.8% of the total variance (PC1 = 20.1%; PC2 = 11.7%).

### 2.4. Pearson’s Correlation Analysis

**Figure 2:**
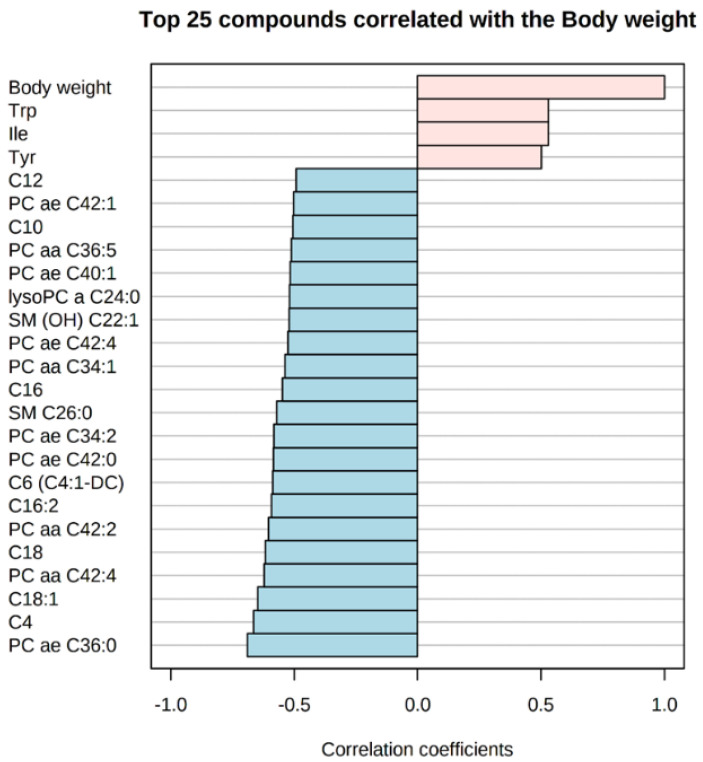
Top 25 significant correlations between BW and liver metabolites of bulls. Blue bars are negative correlations, pink bars are positive correlations.

In the correlation analysis between LW and hepatic metabolites, we found only significant and positive correlation with Symmetric dimethylarginine (SDMA; r = 0.51 and *p* = 0.02). Regarding the correlations between pre-slaughter BW and liver metabolites, we found 29 significant correlations (top 25 in Figure 2). The five strongest correlations found were: Phosphatidylcholine acyl-alkyl C36:0 (PC ae C36:0; r = −0.70 and *p* = 0.001), Butyrylcarnitine (C4; r = −0.66 and *p* = 0.002), Octadecenoylcarnitine (C18: 1; r = −0.64 and *p* = 0.003), Phosphatidylcholine diacyl C42:4 (PC aa C42:4; r = −0.62 and *p* = 0.005), and Octadecanoylcarnitine (C18; r = −0.61 and *p* = 0.006). 

### 2.5. Enrichment Analysis

**Figure 3:**
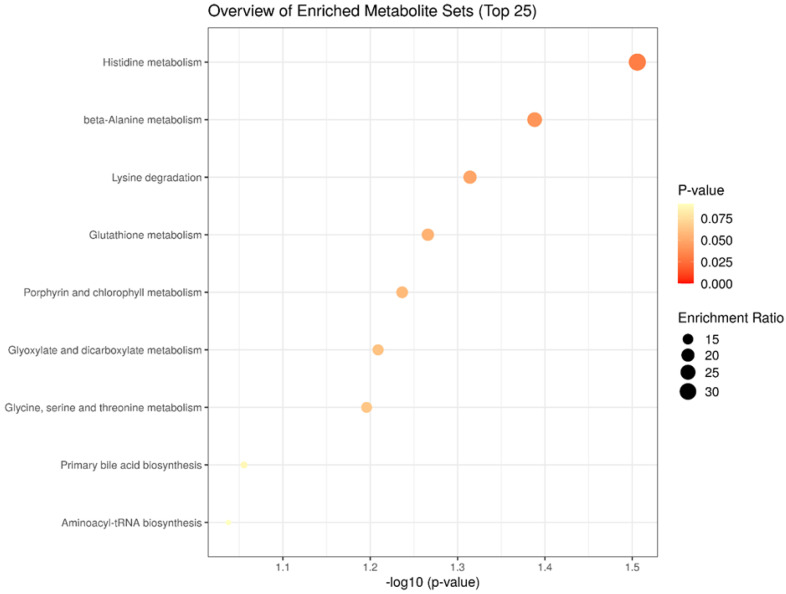
Top biological processes involved with significant liver metabolites of bulls from the three prenatal nutritional treatments (NP, PP, and FP).

In the enrichment analysis of liver metabolites of bulls, we found the top biological processes related to differentially expressed metabolites among the prenatal treatments (Figure 3). Among them, Histidine metabolism (*p* = 0.031), beta-Alanine metabolism (*p* = 0.040), and Lysine degradation (*p* = 0.048) were considered the three most enriched significant metabolic processes.

## 3. Discussion

To our best knowledge and literature search, this is the first study that has assessed the impact of different prenatal supplementation approaches on the liver metabolome of bulls. This innovative and pioneering study significantly contributes to the understanding of the molecular mechanisms involving fetal programming in beef cattle.

During early gestation, the development of some organs is essential for survival, giving priority to receiving the nutrients [21,22]. This nutrient partitioning may reduce the impact of prenatal nutrition on these priority organs (i.e., heart, brain, liver, etc.). It was observed that the higher energy inclusions throughout pregnancy have an impact on the LW of Wagyu cattle fetuses [23]. In our study, LW showed no difference between the treatments, similar to a study conducted by Paradis et al. [24]. These authors observed a none effect of prenatal nutrition (different levels of energy inclusion in the diet during mid-to-late gestation) on the fetal LW of Angus/Simmental cross-breeds; however, they found differences in mRNA abundances of *Longissimus dorsi* muscle and *Semitendinosus* muscle. These findings corroborate that the liver tissue is more resilient than other tissues and organs, such as muscle. 

Regarding to pre-slaughter BW, it was showed that early maternal undernutrition (65% of nutritional requirements) reduced the BW of Pirenaica bulls at slaughter [25]. In a study conducted by Maresca et al. [26], there was no influence of crude protein intake during the third trimester of gestation on BW and growth rate of Angus steers in the finishing phase, similar to what was found in our study. None of these studies [25,26] performed the experiment with the same breed or diet as ours, so many variables can influence the results found. These divergences in the phenotypic responses found in the literature may be related to variables involved during pregnancy (type of supplementation, period in which the dams received the treatment, nutritional level and composition, etc.) and also to breed and genetic factors [1]. Thus, the different prenatal supplementation strategies applied in our study (NP, PP, and FP) showed that higher levels of protein and energy in the diet during entire pregnancy or only in the third trimester in Nellore cows did not change the LW and BW of bulls at slaughter.

We found in the supervised analysis (ANOVA) the main metabolites (Gly, C14:2-OH, Alpha-aaa, Carnosine) related to the different prenatal nutrition strategies in the liver of bulls at slaughter.

The Gly is considered a nonessential amino acid; however, more recently some studies [27,28] showed that the amount of glycine synthesized by the organisms of animals may not be sufficient to fully perform its functions (synthesis of protein, cell signaling, inhibition of calcium influx, antioxidant, inhibitory neurotransmitter in the central nervous system detoxification reactions, anti-inflammation, cytoprotective and immunomodulatory properties, one-carbon metabolism, conjugation with bile acids [29]). Although mild insufficiency of this amino acid does not have serious consequences for survival, a chronic insufficiency can impact on growth, immune responses, and other effects on nutrient metabolism and health [30]. The supplementation during entire pregnancy (FP) led to greater levels of hepatic Gly compared to only the third trimester supplementation (PP). These different levels did not impact on the phenotypes evaluated, but may have affected other traits, mainly related to the immunological and metabolic functions of the bulls.

The C14:2-OH is a long chain acylcarnitine. The components of the acylcarnitine class are a result of an incomplete oxidation of fatty acids and/or amino acids that can be synthesized by mitochondrial and peroxisomal enzymes [31]. Therofore, the circulating levels of acylcarnitines reflects the oxidation rate of fatty acids and amino acids, mainly in liver and skeletal muscle [32,33]. This metabolite class was associated with obesity and type 2 diabetes [33]. Accumulation of acylcarnitines results from a reduction in the ability to oxidize fatty acids and may favor the development of fatty liver disease (triglyceride deposition with or without inflammation, and hypocalcemia; [34]). The levels of acycarnithine and its productive impacts generated in beef cattle are not yet well defined in the literature. However, the different levels of C14:2-OH found between the NP and FP treatments may be related to the different hepatic metabolism of fatty acids. Thus, probably due to the lower rate of complete oxidation of fatty acids, the NP treatment may have presented higher levels of C14:2-OH in relation to the FP treatment.

Alpha-aaa is a metabolite resulting from the oxidation of the amino acid Lysine. The levels of Alpha-aaa has been related to protein oxidation, aging, and disease (renal failure, crystalline sclerosis, diabetes, obesity) in humans [35,36]. This metabolite is considered indicative of oxidative and physiological stress [37]. The supplementation of flax seed to the concentrates offered to steers in the finishing phase had an effect on the levels of Alpha-aaa and other metabolites (Glutamic acid, Methionine, Anserine, and Carnosine [38]). These differentially expressed metabolites altered the flavor, umami, and overall palatability of Longissimus muscle. Studies involving the Alpha-aaa in livestock are scarce; most of them are related to the production of dairy cows, due to the effects of Lysine on milk [39,40]. In our study, protein energy supplementation throughout the gestational period (FP) led to offspring with lower alpha-aaa concentrations. This suggests that FP animals may have greater resilience to hepatic oxidative stress compared to other prenatal treatments.

Carnosine (β-alanyl-L-histidine) is a common dipeptide found in several animal classes (birds, mammals, and fish). This metabolite is found abundantly in beef skeletal muscle [41], being a potent antioxidant [42] with roles related to homeostasis (pH-buffering, muscle ATPase activation to provide energy, metal-ion chelation [43,44]). Carnosine also acts as an epigenetic regulator, increasing histone acetylation in mammalian cell gene expression [45]. In general, the skeletal muscles have the highest concentrations of Carnosine, but liver has greater concentrations than several tissues in beef cattle (e.g., cheek and heart; [46]). Recently, a negative correlation was found between Carnosine and residual feed intake in Nellore cattle [13]. Based only on this metabolite and its functions described in the literature, the higher level of Carnosine found in the NP treatment in relation to the FP treatment would indicate that the greater supply of protein and energy during pregnancy can generate animals with lower feed efficiency. However, complex phenotypes, such as feed efficiency, are controlled by several genetic and biological mechanisms, making it difficult to draw conclusions based only on different levels of one metabolite. 

In the enrichment analysis, we were able to better understand which processes were affected in the liver of bulls at slaughter by the different maternal supplementation strategies. Histidine metabolism, beta-Alanine metabolism, and Lysine degradation were the significant biological pathways related to the treatments (NP, PP, and FP). All significant metabolic processes found are related to amino acid metabolism, therefore implying changes in protein metabolism. The endogenous synthesis of Carnosine is mainly dependent on its “raw materials” (Histidine and beta-Alanine [47]). As plasma levels of Histidine are higher than those of beta-Alanine, the main limiting factor for Carnosine production is the availability of beta-Alanine [48]. Thus, alterations caused by prenatal nutrition in beta-Alanine metabolism may have implicated the different concentrations of hepatic Carnosine found in bulls. The Lysine degradation is strongly related to the different levels of Alpha-aaa discussed above. This significant process corroborates the effect of fetal programming on oxidative metabolism found in some of the differentially expressed metabolites in our study.

We also found some significant correlations between the evaluated phenotypes (LW and pre-slaughter BW) and liver metabolite levels. Values between ±0.40 and ±0.69 are considered moderate correlations [49]. In our study, all significant correlations found were considered to be of moderate magnitude, with the exception of PC ae C36:0. This metabolite showed a strong and negative correlation (r = −0.7) with the pre-slaughter BW. Of the 29 metabolites that showed a significant correlation with pre-slaughter BW, 12 belong to the class of phosphatidylcholines, 9 to acylcarnitines, 3 sphingolipids, 3 amino acids, 1 biogenic amine, and 1 lysophosphatidylcholine. Only amino acids and the biogenic amine showed positive correlation with pre-slaughter BW; the other classes of metabolites showed negative correlations. Regarding LW, the only correlated compound was SDMA (r = 0.51). In a study conducted by Antonelo et al. [50], a weak correlation of slaughter BW with blood creatine kinase (r = 0.23) and albumin (r = −0.24) was found in Nellore cattle. These authors also found a correlation between blood profile (Urea, Creatinine, Albumin, triglycerides, cholesterol, etc.) and other phenotypes (e.g., Longissimus muscle area, backfat thickness, carcass characteristics, etc.). Novais et al. [51] evaluated the serum metabolome correlations in Nellore cattle (high feed efficiency x low feed efficiency) and found several metabolites correlated to the different feed efficiencies (e.g., Progesterone, Retinal, Stearic acid, etc.). Another study was carried out to assess how variability in meat tenderness from Nellore cattle is correlated to *Longissimus thoracis* metabolites [52]. Some metabolites were differentially expressed between the classes (tender and tough; Acetyl-carnitine, adenine, beta-alanine, fumarate, glutamine, and valine), and several metabolites were negatively correlated with WBSF (Warner-Bratzler shear force) values (beta-alanine (r = −0.45), acetyl-carnitine (r = −0.40), fumarate (r = −0.38), valine (r = −0.34), glucose (r = −0.32), glutamine (r = −0.31), and adenine (r = −0.31)). These studies have shown metabolic correlations of two types of tissues with phenotypes of interest in Nellore cattle [51,52]. However, to our knowledge, our study is the first to report correlations between hepatic metabolites and phenotypes in Nellore cattle. Thus, more studies need to be carried out evaluating phenotypic correlations with metabolome in beef cattle, given the scarcity of these types of analysis in the literature. Thus, in our study the BW has a greater influence on the hepatic metabolome than the LW because of the greater magnitudes and quantity of metabolites that showed significance in the analysis.

## 4. Materials and Methods

### 4.1. Experimental Design

A total of 126 Nellore cows and their offspring comprised the study. The cows were fixed-time artificially inseminated with semen from four sires and had their pregnancy diagnosis confirmed 30 days later.

The cows were blocked into three groups of 42 animals based on age, BW, and body condition score (BCS). The three groups were allocated in pasture paddocks of *Brachiaria brizantha* cv. *marandu*, equipped with a trough to supply feed supplement and water. The different prenatal treatments were: NP (control)—Not Programmed, PP—Partial Programming, and FP—Full Programming. NP (control) cows ingested only mineral supplements during the entire pregnancy (0.03% of BW per day). The PP group received protein-energy supplementation (0.3% of BW per day) only in the third trimester of pregnancy, while the FP group received this supplementation (0.3% of BW per day) from the confirmation of pregnancy until delivery. The three groups received mineral supplementation (0.03% of BW, already included in protein-energy supplement formulation: Table 3). Supplements were offered to cows according to average herd weight and adjusted every 14 days until calving. This resulted in estimated consumed values of 0.3% of BW for the energy protein supplement and 0.03% for the mineral supplement. During pregnancy, the paddocks (*Brachiaria brizantha* cv. Marandu) were assessed and presented similar nutritional values: NP (TDN (total digestible nutrients) = 63.07%, CP (crude protein) = 7.38%, and NDF (neutral detergent fiber) = 59.03%), PP (TDN = 64.1%, CP = 7.82, and NDF = 61.43%), and FP (TDN = 61.43%, CP = 7.40%, and NDF = 58.49%). These different prenatal nutrition strategies were chosen based on myogenesis, adipogenesis, and muscle fiber hypertrophy [53] and based on the common supplementation practices used in tropical regions. More details about the pastures, supplements, and the methods used to quantification can be found in [54].

The phenotypic effects of the different prenatal supplementation strategies on dams have been briefly described [56]. After calving, protein-energy supplementation ceased and all offspring (regardless of the nutritional prenatal treatment) were subjected to the same health protocols and nutritional managements, remaining together until weaning (8 ± 1 months). During this period (calving to weaning), the cows received the same mineral supplement (0.03% of BW) as that received during the pregnancy period and remained in an extensive pasture system (paddocks of *Brachiaria brizantha* cv. *Marandu*, as well as during the pregnancy period). 

After weaning, the animals were separated according to sex (males and females), regardless of the treatment, and the males remained until the end of the rearing phase (19 ± 1 months). During the rearing period, young bulls received two types of supplements: in the dry season (winter), an energetic supplement (TDN = 67.55%; CP = 24.78%; NDF = 11.24%; Fat = 2.61%; 0.3% of BW), and throughout the wet season (summer), a protein supplement (TDN = 53.15%; CP = 30.03%; NDF = 9.14%; Fat = 1.65%; 0.1% of BW). From calving to 19 ± 1 months of age, the young bulls remained on pastures of *Brachiaria brizantha* cv. *Marandu* with water ad libitum. 

The 63 bulls started the finishing phase at 19 ± 1 months of age and were slaughtered at 22.5 ± 1 months. During this period, they received three different diets: an adaptation diet (diet 1) provided in the first 15 days (Dry matter (DM) = 48.1%; TDN = 71.0%; CP = 15.0%; NDF = 36.5%; Fat = 3.2%; Dry matter intake (DMI) = 2.21% of BW); a second diet over 35 days (DM = 53.6%; TDN = 73.6%; CP = 14.0%; NDF = 31.1%; Fat = 3.4%; DMI = 2.20% of BW); and a third diet for 56 days (DM = 60.6%; TDN = 76.2%; CP = 13.0%; NDF = 25.8%; Fat = 3.7%; DMI = 2.04% of BW). At the end of the finishing phase, the animals were slaughtered at the FZEA/USP school slaughterhouse, located approximately 500 m from the feedlot installations. The slaughter and processing of the carcasses were carried out in accordance with the procedures required by the Ministry of Agriculture, Livestock, and Supply of Brazil (MAPA, Normative Instruction No. 9 of 2004).

### 4.2. Phenotypes, Liver Sample Collection and Preparation

The 63 bulls were weighted at 22.5 ± 1 months of age (pre-slaughter BW) with a Coimma^®^ (Dracena—SP, Brazil) digital scale. All animals were slaughtered, and the liver was weighed and collected for metabolomics analysis. The organ was sampled (distal portion of left lobe) and immediately stored in liquid nitrogen, and finally kept in an ultrafreezer (−80 °C) until extraction. From the 63 male offspring, we selected 18 (6 per treatment randomly) for liver metabolomics analysis. We performed the extraction of liver using an extraction solvent composed by 85 mL ethanol (HPLC grade) and 15 mL of phosphate buffer (0.01 M, pH = 7.5 at 25 °C). All procedures were carried out below 0 °C, using dry-ice to avoid degeneration processes. The samples were weighed and homogenized via a bead-based homogenizer (20 s at 5500 rpm) using the extraction solvent described. This homogenization process was repeated three times, followed by centrifuge (10,000× *g* for 5 min). The supernatant was transferred into a tube and immediately stored in an ultrafreezer until metabolite quantification (AbsoluteIDQ^®^ p180 Kit, Biocrates Life Sciences AG, Innsbruck, Austria). More details about the extraction protocol can be found in [57].

### 4.3. Targeted Metabolomics

The Apex Science company carried out the metabolomics analysis (Campinas, São Paulo, Brazil). Metabolites were quantified using AbsoluteIDQ^®^ p180 Kit (Biocrates Life Sciences AG, Innsbruck, Austria). The kit covers 188 metabolites, of which 21 are amino acids, 21 biogenic amines, 40 acylcarnitines (Cx:y), 14 lysophosphatidylcholines (lysoPC), 76 phosphatidylcholines (PC), and 15 sphingolipids (SMx:y). The x and y in these formulas represent the number of carbons and double bonds of all chains, respectively. The amino acids and biogenic amines were derivatized using phenylisothiocyanate. These metabolite classes were analyzed by liquid chromatography tandem-mass spectrometry (HPLC-MS/MS) using an AB SCIEX 4000 QTrap mass spectrometer (AB SCIEX, Darmstadt, Germany) with electrospray ionization. The lysophosphatidylcholines, phosphatidylcholines, acylcarnitines, and hexose were performed by flow injection analysis-tandem mass spectrometry (FIA-MS/MS).

MetIDQ^®^ software v1.0 (part of the AbsoluteIDQ^®^ p180 kit) was used to perform the data analysis for metabolite quantification and quality assessment. Metabolite concentrations (measured in μM) were calculated using internal standards. Biocrates experimentally determines the metabolite-specific limits of detection (LOD) of the assay.

### 4.4. Statistical Analysis

Data processing and univariate analysis (analysis of variance; ANOVA) of metabolites and phenotypes were performed using the R software environment (version 4.1.2). Metabolites with more than 70% of samples below LOD or with the same values among samples were removed from the dataset (180 metabolites remaining). The LOD values that remained in the metabolome after filtering were replaced by the mean of each variable. The model was implemented through the “LM” function in R.

The statistical model used for the bulls’ metabolomic and phenotypic analyses:

(1) $$Y_{\mathrm{jk}}=\;\mu +{\;\beta }_{1}{\mathrm{Age}}_{b1}+{\;\mathrm{Treat}}_{j}+e_{\mathrm{jk}}\;$$

where Y_jk_ is the observed metabolite/phenotype from the kth animal, recorded on jth treatment; μ is just a constant; β_1_ is the regression coefficient of covariate animal’s age; Age_b1_ is the observed value for bull’s age of the kth animal; Treat_j_ is the fixed effect of the jth treatment; and e_jk_ is the residual random term. The residuals were tested for normality (Shapiro–Wilk test) and for homoscedasticity (Levene’s test), and the differences between treatments were considered significant when *p* ≤ 0.05 by the Tukey–Kramer test. 

In addition, the metabolite concentration table was uploaded to MetaboAnalyst 5.0 [58], and the data were auto-scaled (mean-centered and divided by the standard deviation of each variable) before analysis. We performed a principal component analysis (PCA), an enrichment analysis, and a correlation analysis. PCA was performed to assess the clusters between treatments (NP, PP, and FP). The enrichment analysis was carried out by a MetaboaAnalyst to identify the most relevant biological processes associated with the differentially expressed metabolites (identified in univariate analysis) based on the Kyoto Encyclopedia of Genes and Genomes database (KEGG Pathway). This database allows one to find different biological pathways (e.g., carbohydrate metabolism, energy metabolism, lipid metabolism, nucleotide metabolism, amino acid metabolism) related to the input (differentially expressed metabolites). Biological processes with *p* ≤ 0.05 were considered significant. The PatternHunter tool on MetaboAnalyst was used for finding the correlations between the phenotypes (pre-slaughter BW and LW) and metabolites. In this method, we use Pearson’s correlation as a distance measure. The correlations were considered significant when *p* ≤ 0.05.

## 5. Conclusions

The different prenatal supplementations did not impact on the phenotypes evaluated (LW and pre-slaughter BW). However, maternal nutrition had an effect on the liver metabolome of Nellore bulls at slaughter. The differentially-expressed metabolites and biological processes were found to mainly affect oxidative metabolism. The Pearson’s correlation analysis revealed a greater association of pre-slaughter BW with the hepatic metabolome than LW, due to the magnitude and amount of associated metabolites. 

## Figures and Tables

**Table 1:** Phenotypes (pre-slaughter BW and LW) assessed in the bulls from the different prenatal treatments (NP, PP, and FP; mean ± standard deviation) with their respective *p*-values and standard errors of mean (SEM).

Traits (kg)	NP	PP	FP	SEM	*p* Value
BW	591.2 ± 40.05	602.6 ± 49.65	597.4 ± 51.06	5.858	0.685
LW	7.227 ± 0.734	7.295 ± 0.779	7.286 ± 0.714	0.092	0.924

NP—not programmed; PP—partial programming; FP—full programming; SEM—Standard error of the mean.

**Table 2:** Liver significant metabolites (µM; mean ± standard deviation) of bulls submitted to the different prenatal nutrition approaches (NP, PP, and FP) with their respective *p*-values and standard errors of mean (SEM).

Metabolite	NP	PP	FP	SEM	*p* Value
Gly	1232 ± 151.5 ^ab^	1099 ± 86.60 ^a^	1325 ± 187.3 ^b^	57.91	0.024
C14:2-OH	0.006 ± 0.004 ^a^	0.002 ± 0.005 ^ab^	0.001 ± 0.001 ^b^	0.001	0.042
Alpha-aaa	8.155 ± 2.106 ^a^	8.432 ± 3.799 ^a^	4.457 ± 1.773 ^b^	1.045	0.043
Carnosine	122.6 ± 29.84 ^a^	107.3 ± 27.33 ^ab^	84.36 ± 12.27 ^b^	9.451	0.050

The small letters represent the significant contrasts. NP—not programmed; PP—partial programming; FP—full programming; SEM—Standard error of the mean.

**Table 3:** Ingredients and nutrients content of the dams’ supplement.

**Ingredients (Dry Matter)**	**Mineral Supplement**	**Protein-Energy Supplement**
Corn (%)	35.00	60.00
Soybean meal (%)	-	30.00
Dicalcium phosphate (%)	10.00	-
Urea 45% (%)	-	2.50
Salt (%)	30.00	5.00
Minerthal 160 MD (%) *	25.00	2.50
**Nutrients**	**Mineral Supplement**	**Protein-Energy Supplement**
Total digestible nutrients (%)	26.76	67.55
Crude protein (%)	2.79	24.78
Non-protein nitrogen (%)	-	7.03
Acid detergent fiber (%)	1.25	4.76
Neutral detergent fiber (%)	4.29	11.24
Fat (%)	1.26	2.61
Calcium (g/kg)	74.11	6.20
Phosphorus (g/kg)	59.38	7.24

* Mineral premix composition (Minerthal company): Calcium = 8.6 g/kg; Cobalt = 6.4 mg/kg; Copper = 108 mg/kg; Sulfur = 2.4 g/kg; Fluorine = 64 mg/kg; Phosphorus = 6.4 g/kg; Iodine = 5.4 mg/kg; Manganese = 108 mg/kg; Selenium = 3.2 mg/kg; Zinc = 324 mg/kg; Sodium monensin = 160 mg/kg [55].

## Data Availability

The datasets generated during and/or analyzed during the current study are available from the corresponding author on reasonable request [due to restrictions on privacy].

## Supplementary Materials

The following supporting information can be downloaded at: https://www.mdpi.com/article/10.3390/metabo12050441/s1, Table S1: Liver metabolites (µM; mean ± standard deviation) of bulls submitted to the different prenatal nutrition approaches (NP, PP, and FP) with their respective *p* values.
